# A novel mRNA-miRNA-lncRNA competing endogenous RNA triple sub-network associated with prognosis of pancreatic cancer

**DOI:** 10.18632/aging.101933

**Published:** 2019-05-06

**Authors:** Wenlong Wang, Weiyang Lou, Bisha Ding, Beng Yang, Hongda Lu, Qingzhi Kong, Weimin Fan

**Affiliations:** 1 Intensive Care Unit, Hangzhou Hospital of Traditional Chinese Medicine, Zhejiang Province, Hangzhou 310007, China; 2 Clinical College of Traditional Chinese Medicine, Hubei University of Traditional Chinese Medicine, Hubei Province, Wuhan 430065, China; 3 Program of Innovative Cancer Therapeutics, Division of Hepatobiliary and Pancreatic Surgery, Department of Surgery, First Affiliated Hospital, College of Medicine, Zhejiang University, Key Laboratory of Organ Transplantation, Zhejiang Province, Hangzhou 310003, China; 4 Key Laboratory of Organ Transplantation, Zhejiang Province, Hangzhou 310003, China; 5 Key Laboratory of Combined Multi-organ Transplantation, Ministry of Public Health, Hangzhou 310000, China; 6 The Central Hospital of Wuhan, Tongji Medical College, Huazhong University of Science and Technology and Wuhan City Oncology Institute, Hubei Province, Wuhan 430014, China; * Equal contribution

**Keywords:** pancreatic cancer, bioinformatic analysis, competing endogenous RNA (ceRNA), prognosis, long noncoding RNA (lncRNA), microRNA (miRNA)

## Abstract

Background: Recently, increasing evidence has uncovered the roles of mRNA-miRNA-lncRNA network in multiple human cancers. However, a systematic mRNA-miRNA-lncRNA network linked to pancreatic cancer prognosis is still absent. Methods: Differentially expressed genes (DEGs) were first identified by mining GSE16515 and GSE15471 datasets. DAVID database was utilized to conduct functional enrichment analysis. Protein-protein interaction (PPI) network was built using STRING database, and hub genes were identified by Cytoscape plug-in CytoHubba. Upstream miRNAs and lncRNAs of mRNAs were predicted by miRTarBase and miRNet, respectively. Expression, survival and correlation analysis for genes, miRNAs and lncRNAs were performed *via* GEPIA, Kaplan-Meier plotter and starBase. Results: 734 and 180 upregulated and downregulated significant DEGs were identified, respectively. Functional enrichment analysis revealed that they were significantly enriched in focal adhesion, pathways in cancer and metabolic pathways. According to node degree, hub genes in the PPI networks were screened, such as TGFB1 and ALB. Among the top 20 hub genes, 7 upregulated genes and 2 downregulated hub genes had significant prognostic values in pancreatic cancer. 33 miRNAs were predicted to target the 9 key genes. But only high expression of 8 miRNAs indicated favorable prognosis in pancreatic cancer. Then, 90 lncRNAs were predicted to potentially bind to the 8 miRNAs. SCAMP1, HCP5, MAL2 and LINC00511 were finally identified as key lncRNAs. By combination of results from expression, survival and correlation analysis demonstrated that MMP9/ITGB1-miR-29b-3p-HCP5 competing endogenous RNA (ceRNA) sub-network was linked to prognosis of pancreatic cancer. Conclusions: In a word, we established a novel mRNA-miRNA-lncRNA sub-network, among which each RNA may be utilized as a prognostic biomarker of pancreatic cancer.

## Introduction

Pancreatic cancer ranks as the third leading cause of cancer-related deaths in the United States [1]. Pancreatic cancer is one of the most quickly fatal cancers all over the world, with mortality almost equal to its incidence [2]. Besides, pancreatic cancer lacks of obvious early symptoms, thereby leading to most patients with pancreatic cancer being diagnosed at advanced stages. Over the past years, in spite of huge improvements in surgery, chemotherapy and radiotherapy for pancreatic cancer have been achieved, prognosis is still extremely dismal with nearly 100% of 5-year mortality rate [2,3]. Furthermore, to date, the precise mechanisms how pancreatic cancer occurs and progresses are still not clearly elucidated. It is essential to explore underlying molecular mechanisms and develop effective therapeutic targets and novel prognostic biomarkers for pancreatic cancer.

In 2011, Salmena *et al.* proposed a novel regulatory mechanism between noncoding RNA (ncRNA) and messenger RNA (mRNA), namely competing endogenous RNA (ceRNA) hypothesis [4]. In this theory, cross-talk between ceRNAs achieves by competitively binding to shared miRNAs [5]. The discovery of ceRNA mechanism has attracted the attention of many researchers and scholars. They conducted a variety of related investigations in respective study field, including cancer. Long non-coding RNAs (lncRNAs) are a class of ncRNA with length more than 200 nucleotides, which have been reported to act as miRNA sponges to decrease miRNA abundance, thus relieving inhibitory effect of miRNA on downstream target genes [6–9]. Increasing evidence has well documented that lncRNA-miRNA-mRNA ceRNA network plays key roles in multiple human cancers, such as breast cancer [10], gastric cancer [11], liver cancer [12] as well as pancreatic cancer [13]. However, current knowledge for lncRNA-miRNA-mRNA in human cancers is not enough, including pancreatic cancer.

In this study, we first acquired aberrantly expressed mRNAs by mining two GEO datasets. Subsequently, we conducted functional enrichment analysis for these aberrantly expressed mRNAs. Then, protein-protein interaction analysis was also employed, and hub genes were identified. By combining expression and prognostic roles of hub genes in pancreatic cancer, 7 upregulated genes and 2 downregulated genes were selected for subsequent analysis. Next, upstream miRNAs and lncRNAs were predicted. Besides, we also further evaluated the prognostic roles of these miRNAs and lncRNAs in pancreatic cancer. The correlations of mRNAs, miRNAs and lncRNAs were also determined. Finally, a novel ceRNA regulatory sub-network associated with pancreatic cancer patients’ prognosis was successfully established. Intriguingly, each RNA in this ceRNA network may be utilized to indicate prognosis of pancreatic cancer based on our current analytic results. They may also serve as promising diagnostic biomarkers or therapeutic targets for pancreatic cancer in the future.

## RESULTS

### Screening of significant DEGs in pancreatic cancer

**Figure 1:**
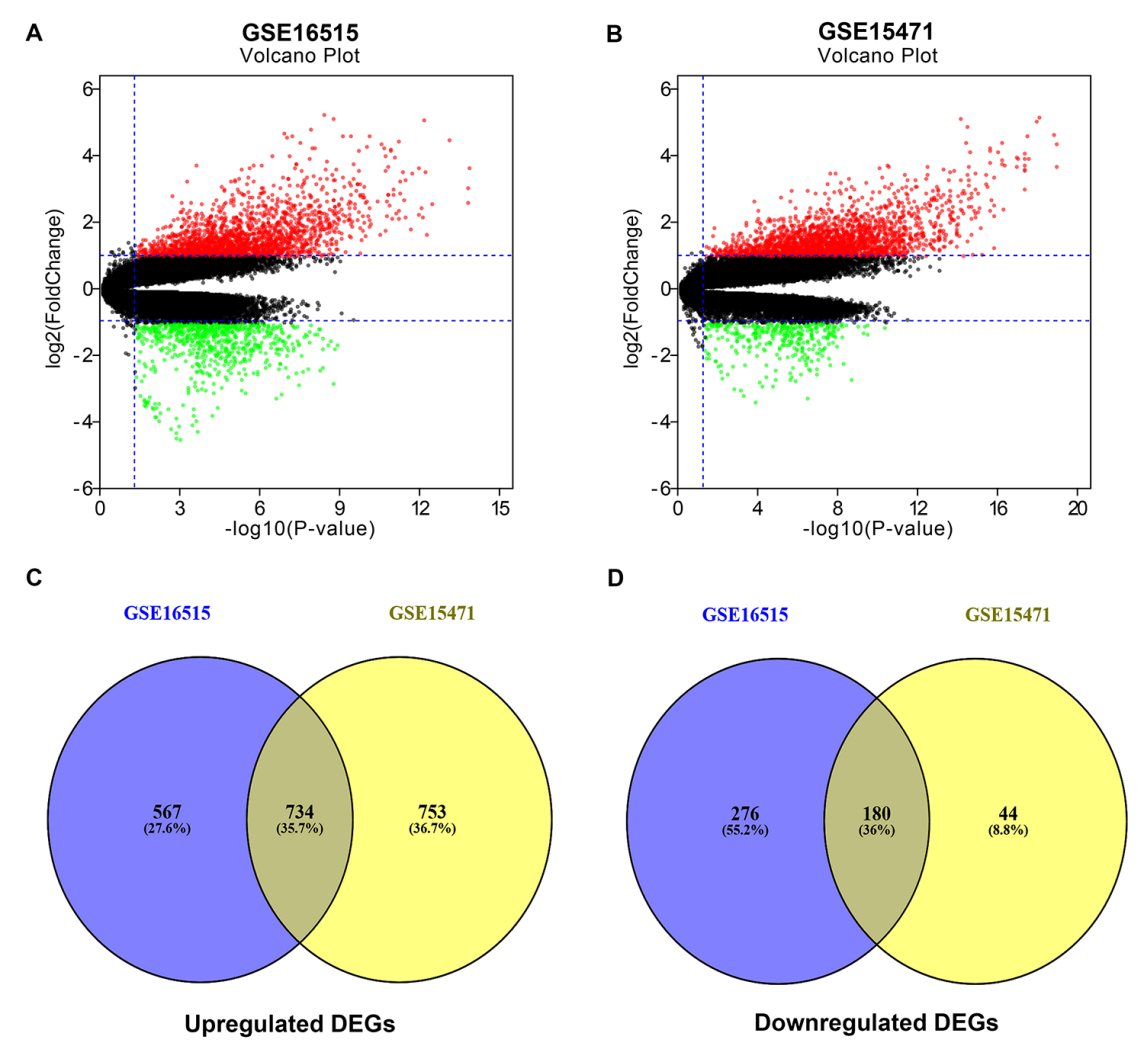
**Identification of significant differentially expressed genes (DEGs) in pancreatic cancer.** (**A**) Volcano plot showing the DEGs identified from GSE16515. (**B**) Volcano plot showing the DEGs identified from GSE15471. X axis represents log transformed P value, and Y axis indicates the mean expression differences of genes between pancreatic cancer samples and normal samples. Note: The two volcano plots showed all of the DEGs; the black dots represent genes that are not differentially expressed between pancreatic cancer samples and normal samples, and the green dots and red dots represent the downregulated and upregulated genes in pancreatic cancer samples, respectively. |log_2_FC| >1 and adj. p-value < 0.05 were set as the cut-off criteria. (**C**) The intersection of upregulated DEGs of GSE16515 and GSE15471 datasets. (**D**) The intersection of downregulated DEGs of GSE16515 and GSE15471 datasets. The intersected DEGs were defined as the significant DEGs.

In search of gene expression microarrays regarding pancreatic cancer in the GEO database, two datasets (GSE16515 and GSE15471) were finally included. Subsequently, differential expression analysis was conducted by using GEO2R (|log_2_FC| > 1 and adj. p-value < 0.05), and some DEGs in each dataset were discovered. These DEGs from GSE16515 and GSE15471 datasets were shown in Figure 1A and Figure 1B, respectively. Next, we further identified some significant DEGs which were commonly appeared in the two datasets. As shown in Figure 1C and Figure 1D, a total of 734 and 180 upregulated and downregulated significant DEGs in pancreatic cancer were identified. These upregulated and downregulated significant DEGs were concretely listed in Table S1 and Table S2, respectively. Also, these significant DEGs were selected for subsequent analyses.

### Functional enrichment analysis for the significant DEGs

To predict the underlying biological function and corresponding pathways of these significant DEGs, DAVID database was introduced to perform functional enrichment analysis, including three GO terms (BP: biological process; CC: cellular component; MF: molecular function) and KEGG pathway.

**Figure 2:**
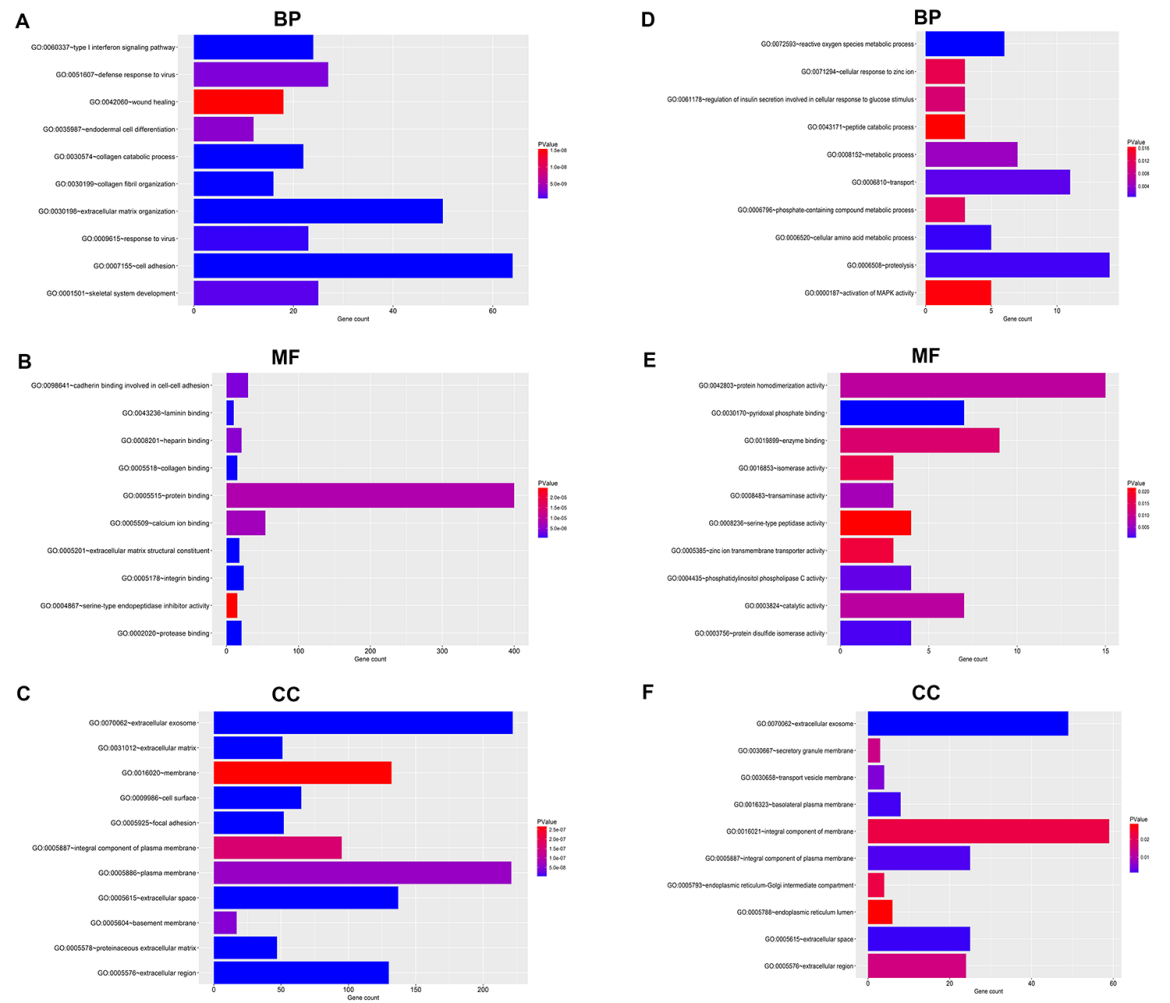
**GO functional annotation for the significant DEGs.** (**A**) The top ten enriched biological process (BP) of the upregulated significant DEGs. (**B**) The top ten enriched molecular function (MF) of the upregulated significant DEGs. (**C**) The top ten enriched cellular component (CC) of the upregulated significant DEGs. (**D**) The top ten enriched biological process (BP) of the downregulated significant DEGs. (**E**) The top ten enriched molecular function (MF) of the downregulated significant DEGs. (**F**) The top ten enriched cellular component (CC) of the downregulated significant DEGs.

**Figure 3:**
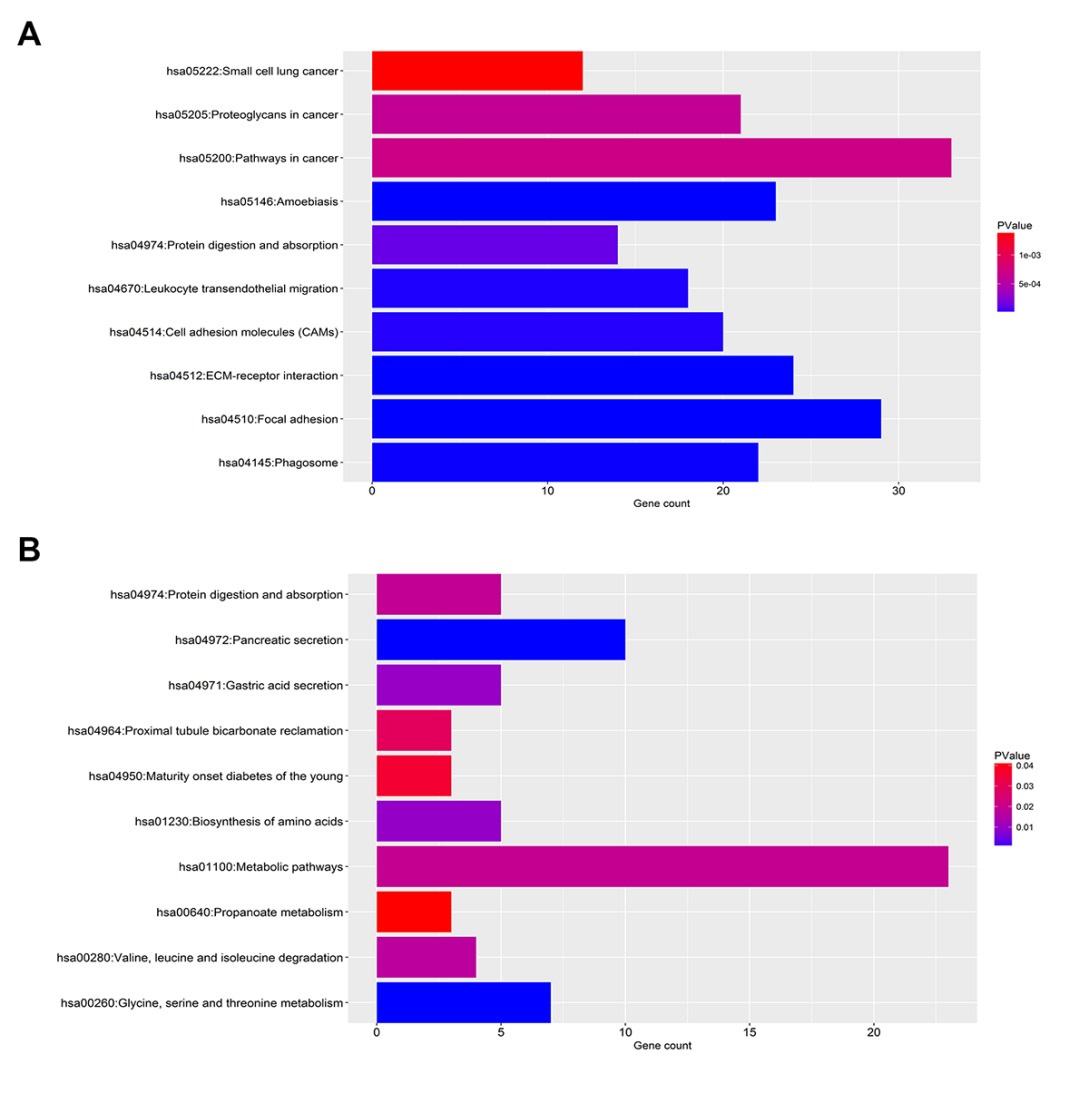
**KEGG pathway enrichment analysis for the significant DEGs.** (**A**) The top ten enriched KEGG pathways of the upregulated significant DEGs. (**B**) The top ten enriched KEGG pathways of the downregulated significant DEGs.

For upregulated significant DEGs, as presented in Figure 2A-C, the enriched GO functions included cell adhesion, extracellular matrix organization and wound healing in the BP category; protein binding, calcium ion binding and cadherin binding involved in cell-cell adhesion in the MF category; and extracellular exosome, plasma membrane and membrane in the CC category. Besides, Figure 3A revealed that these upregulated significant DEGs were significantly enriched in some cancer-associated pathways, such as pathways in cancer, focal adhesion, proteoglycans in cancer and small cell lung cancer.

As shown in Figure 2D-E, the enriched GO functions for downregulated significant DEGs included proteolysis, transport and metabolic process in the BP category; protein homodimerization activity, enzyme binding and pyridoxal phosphate binding in the MF category; and integral component of membrane, extracellular exosome and integral component of plasma membrane in the CC category. Similarly, some enriched KEGG pathways were also observed, among which metabolic pathways, pancreatic secretion and glycine, serine and threonine metabolism were the most highly enriched pathways (Figure 3B).

### Establishment and analysis of PPI network

**Figure 4:**
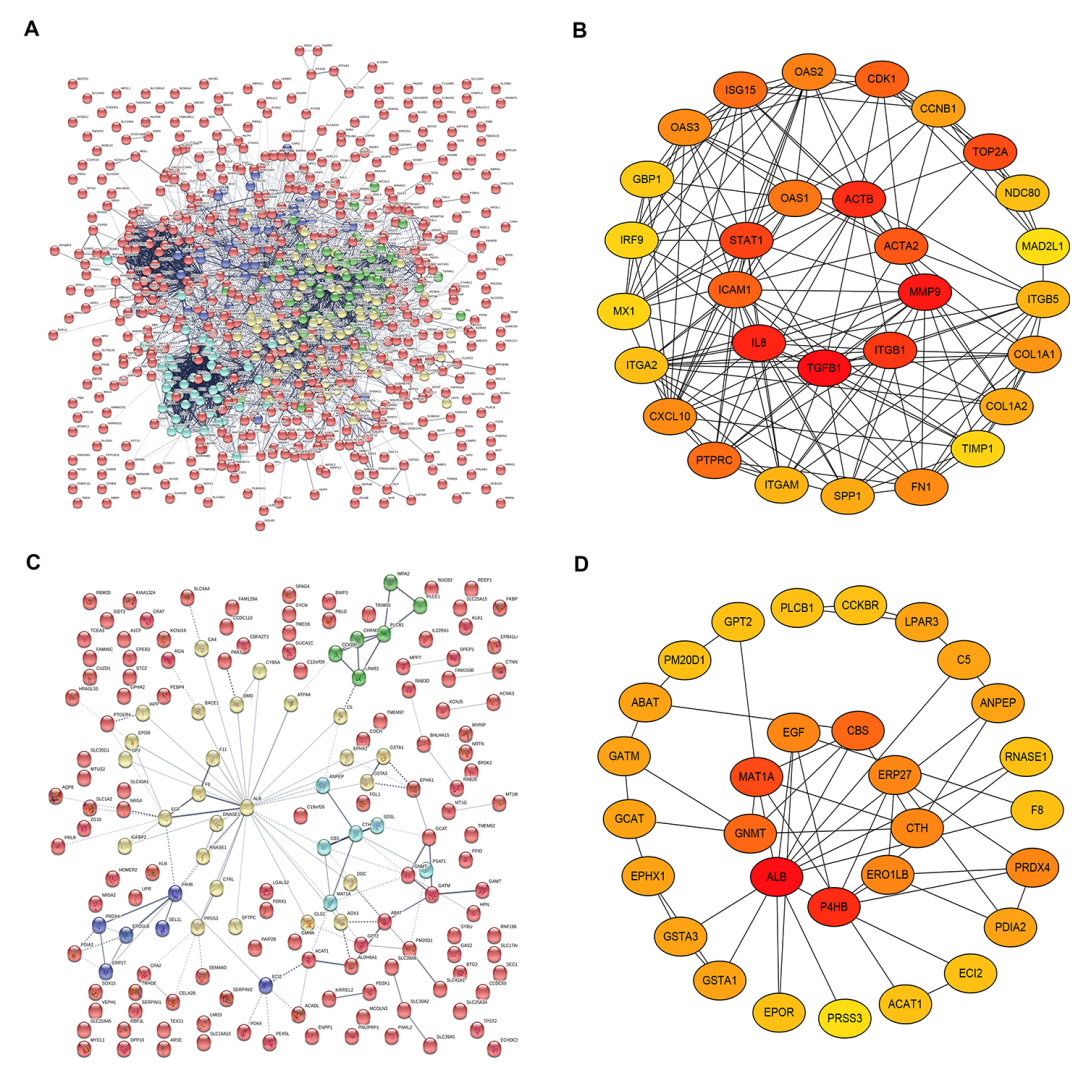
**The top 30 hub genes identified in protein-protein interaction (PPI) networks.** (**A**) The PPI network of the significant upregulated DEGs. (**B**) The top 30 hub genes of the significant upregulated DEGs. (**C**) The PPI network of the significant downregulated DEGs. (**D**) The 30 hub genes of the significant downregulated DEGs.

On the basis of the data from STRING database analysis, PPI networks of the upregulated significant DEGs and downregulated significant DEGs were constructed as shown in Figure 4A and Figure 4C, respectively. According to node degree, we identified some hub genes among these significant DEGs. For better visualization, the interactors of top 30 upregulated (Figure 4B) and downregulated (Figure 4D) hub genes were re-built using Cytoscape software. Additionally, the top 30 hub genes and their corresponding node degrees were listed in Table 1 and top 10 upregulated hub genes were TGFB1, MMP9, CXCL8 (IL8), ACTB, ITGB1, STAT1, TOP2A, ACTA2, ICAM1 and CDK1, and top 10 downregulated hub genes were ALB, EGF, P4HB, MAT1A, GNMT, ABAT, CBS, CTH, PRSS3 and ECI2. The 20 hub genes were chosen for following analyses.

**Table 1:** The top 30 hub genes in PPI networks.

Upregulated gene		Downregulated gene	
Gene symbol	Degree	Gene symbol	Degree
TGFB1	94	ALB	28
MMP9	78	EGF	10
CXCL8 (IL8)	75	P4HB	8
ACTB	70	MAT1A	6
ITGB1	67	GNMT	6
STAT1	65	ABAT	6
TOP2A	64	CBS	6
ACTA2	58	CTH	6
ICAM1	57	PRSS3	5
CDK1	57	ECI2	5
PTPRC	56	PLCB1	5
ISG15	56	ERP27	4
OAS1	55	PM20D1	4
OAS2	53	PRDX4	4
FN1	52	GCAT	4
CXCL10	52	LPAR3	4
OAS3	52	CCKBR	4
COL1A1	51	EPHX1	4
CCNB1	47	ACAT1	4
SPP1	46	ERO1LB	4
COL1A2	46	GATM	4
ITGB5	45	EPOR	3
ITGAM	45	GPT2	3
ITGA2	43	RNASE1	3
NDC80	43	PDIA2	3
GBP1	42	ANPEP	3
IRF9	41	C5	3
MX1	41	AOX1	3
TIMP1	41	SDSL	3
HLA-A	40	CHRM3	3

### Identification of key genes in pancreatic cancer

**Figure 5:**
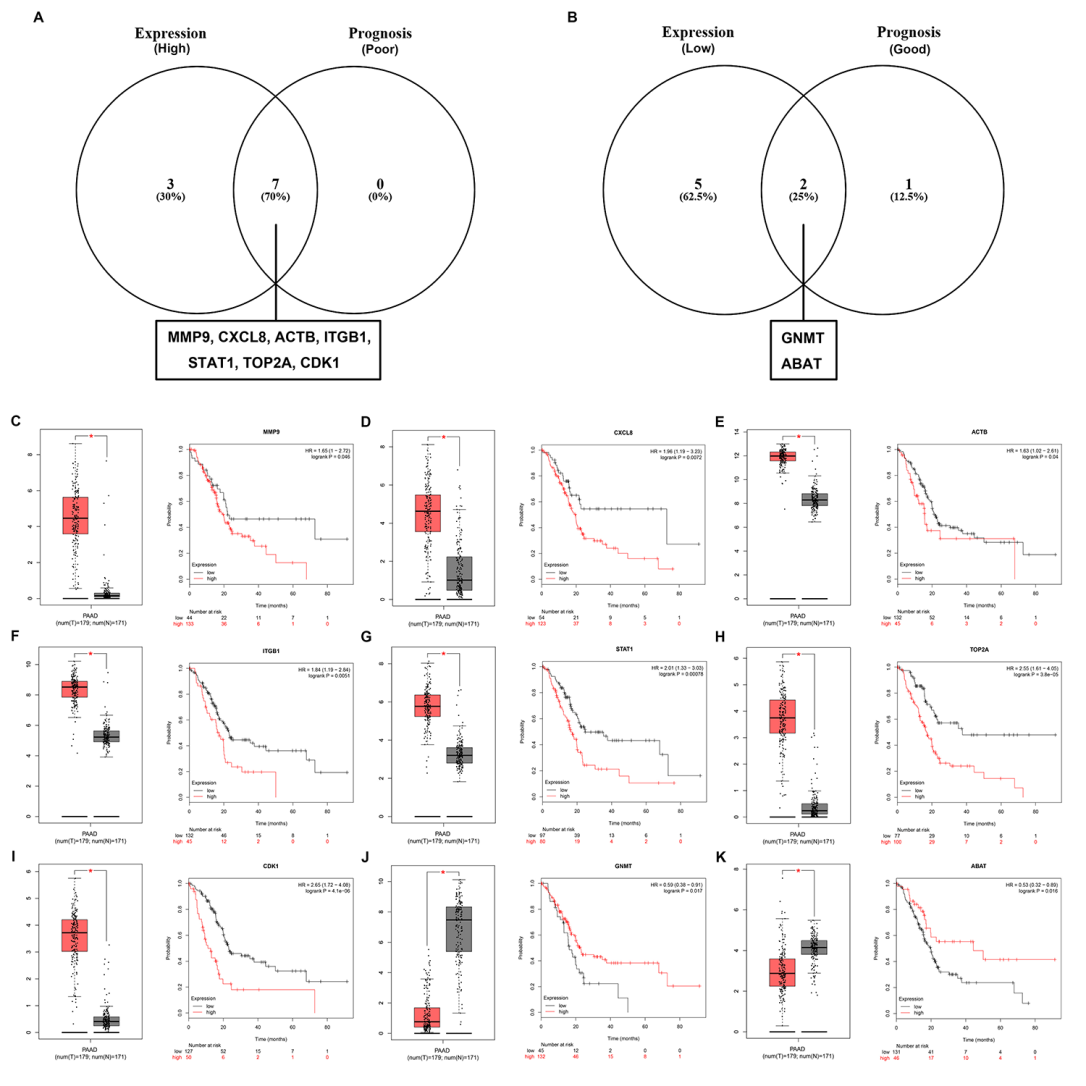
**Screening the key genes in pancreatic cancer.** (**A**) Identification of key genes among the top 10 hub genes of the significant upregulated DEGs by combining expression and prognosis analyses using GEPIA and Kaplan Meier-plotter databases, respectively. (**B**) Identification of key genes among the top 10 hub genes of the significant downregulated DEGs by combining expression and prognosis analyses using GEPIA and Kaplan Meier-plotter databases, respectively. (**C**) Expression and prognostic value of MMP9 in pancreatic cancer. (**D**) Expression and prognostic value of CXCL8 in pancreatic cancer. (**E**) Expression and prognostic value of ACTB in pancreatic cancer. (**F**) Expression and prognostic value of ITGB1 in pancreatic cancer. (**G**) Expression and prognostic value of STAT1 in pancreatic cancer. (**H**) Expression and prognostic value of TOP2A in pancreatic cancer. (**I**) Expression and prognostic value of CDK1 in pancreatic cancer. (**J**) Expression and prognostic value of GNMT in pancreatic cancer. (**K**) Expression and prognostic value of ABAT in pancreatic cancer.

In order to further identify key genes in pancreatic cancer, we determined the expression and prognostic values of the top 10 upregulated and downregulated hub genes using GEPIA and Kaplan-Meier plotter databases, respectively. Combined the results of expression analysis and survival analysis, we found that 7 upregulated hub genes (MMP9, CXCL8, ACTB, ITGB1, STAT1, TOP2A and CDK1) were not only significantly upregulated in pancreatic cancer samples but also the increased expression of the 7 genes indicated poor prognosis in patients with pancreatic cancer (Figure 5A and Figure 5C-I), and 2 downregulated hub genes (GNMT and ABAT) were commonly appeared in “low expression” gene set and “good prognosis” gene set (Figure 5B and Figure 5J-K). In next analyses, we are interested to investigate the 9 key genes, including 7 upregulated hub genes and 2 downregulated hub genes.

### Prediction and validation of upstream key miRNAs of key genes

**Figure 6:**
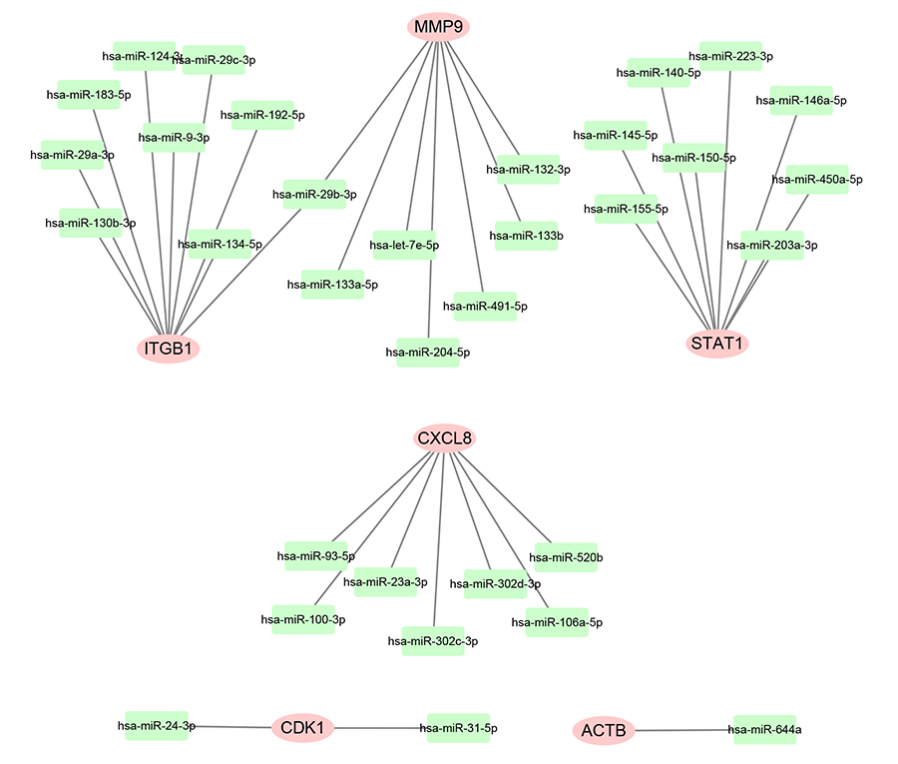
**Construction of miRNA-gene network using Cytoscape software.**

**Figure 7:**
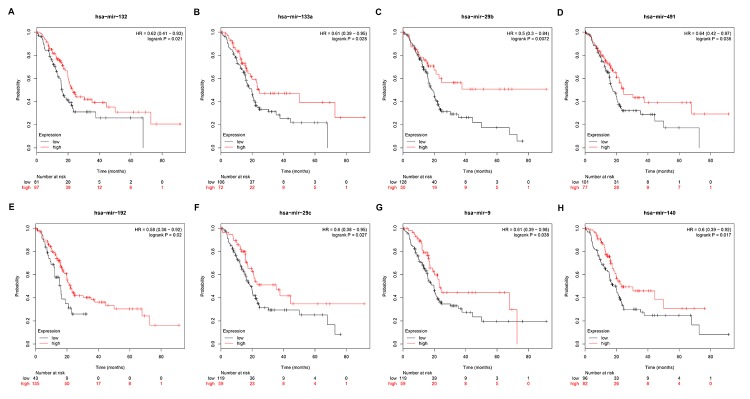
**Prognostic values of miRNAs in pancreatic cancer.** (**A**) Prognostic value of has-miR-132 in pancreatic cancer. (**B**) Prognostic value of has-miR-133a in pancreatic cancer. (**C**) Prognostic value of has-miR-29b in pancreatic cancer. (**D**) Prognostic value of has-miR-491 in pancreatic cancer. (**E**) Prognostic value of has-miR-192 in pancreatic cancer. (**F**) Prognostic value of has-miR-29c in pancreatic cancer. (**G**) Prognostic value of has-miR-9 in pancreatic cancer. (**H**) Prognostic value of has-miR-140 in pancreatic cancer.

Subsequently, we predicted upstream miRNAs of the 9 key genes by using an experimentally validated microRNA-target gene interactions database, miRTarBase. As mentioned above, in this study, we only included microRNA-target gene interactions that were validated by reporter assay. Finally, we identified a total of 33 miRNAs that could potentially regulate six key genes (ITGB1, MMP9, STAT1, CXCL8, CDK1 and ACTB) expression as presented in Figure 6 and Table S3. Upstream potential miRNAs of three other key genes (TOP2A, GNMT and ABAT) were not observed. Besides, we noticed that all the six key genes were upregulated hub genes, with unfavorable prognostic values in pancreatic cancer. Based on the classical inverse relationship between miRNA and target gene, we hypothesized that the upstream miRNAs of 6 upregulated key genes should theoretically display favorable prognostic roles. Therefore, we further assessed prognostic values of the 33 predicted miRNAs using Kaplan-Meier plotter database. The results of survival analysis showed that 8 (miR-132-3p, miR-133a-5p, miR-29b-3p, miR-491-5p, miR-192-5p, miR-29c-3p, miR-9-3p and miR-140-5p) out of 33 miRNAs functioned as positive prognostic biomarkers for patients with pancreatic cancer as presented in Figure 7. The 8 miRNAs were defined as the key miRNAs.

### Prediction and validation of upstream key lncRNAs of key miRNAs

**Figure 8:**
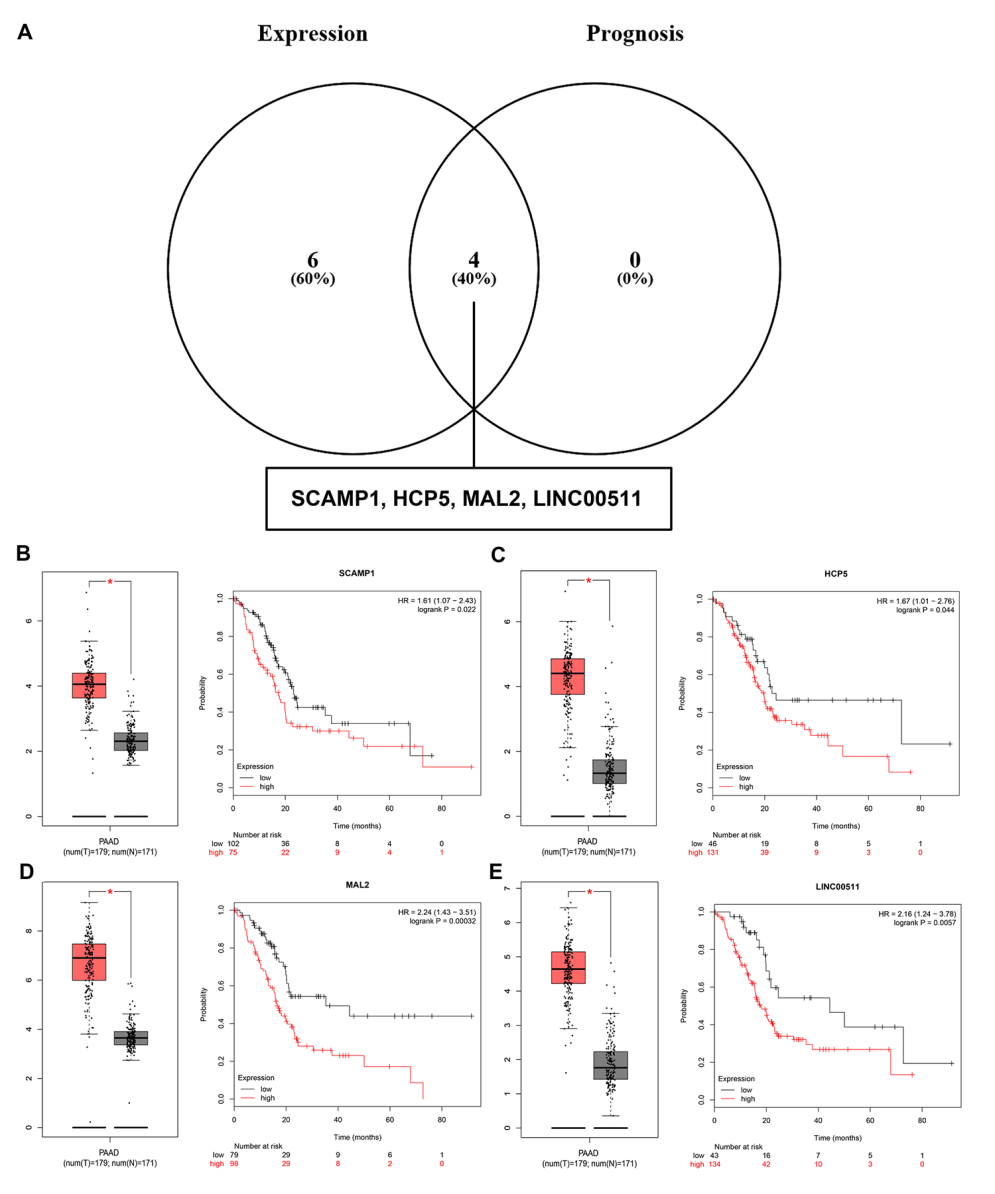
**Screening the key lncRNAs in pancreatic cancer.** (**A**) Identification of key lncRNAs among the predicted lncRNAs by combining expression and prognosis analyses using GEPIA and Kaplan Meier-plotter databases, respectively. (**B**) Expression and prognostic value of SCAMP1 in pancreatic cancer. (**C**) Expression and prognostic value of HCP5 in pancreatic cancer. (**D**) Expression and prognostic value of MAL2 in pancreatic cancer. (**E**) Expression and prognostic value of LINC00511 in pancreatic cancer.

Growing studies have suggested that lncRNA functions as ceRNA to interact with mRNA by competing for shared miRNA [14,15]. In view of this theory, we further predicted those lncRNAs that can potentially bind to the 8 key miRNAs (miR-132-3p, miR-133a-5p, miR-29b-3p, miR-491-5p, miR-192-5p, miR-29c-3p, miR-9-3p and miR-140-5p) using an online database miRNet. A total of 90 lncRNAs were discovered (Table S4). There is a negative correlation between lncRNA and miRNA based on the ceRNA hypothesis. Thus, we analyzed these lncRNAs expression in pancreatic cancer using GEPIA database. Only 10 (SCAMP1, EMG1, HCP5, TUG1, MAL2, H19, LINC00511, RP11-311C24.1, RP11-400F19.6 and CTC-459F4.3) out of 90 lncRNAs were significantly upregulated in pancreatic cancer samples when compared with normal controls. Subsequent survival analysis for the 10 upregulated lncRNAs demonstrated that patients with high expression of SCAMP1, HCP5, MAL2 and LINC00511 had unfavorable prognosis. Combined the results of expression analysis and survival analysis for these predicted lncRNAs, we re-defined the 4 lncRNAs (SCAMP1, HCP5, MAL2 and LINC00511) as key lncRNAs (Figure 8).

### Construction of key mRNA-miRNA -lncRNA triple sub-network in pancreatic cancer

**Figure 9:**
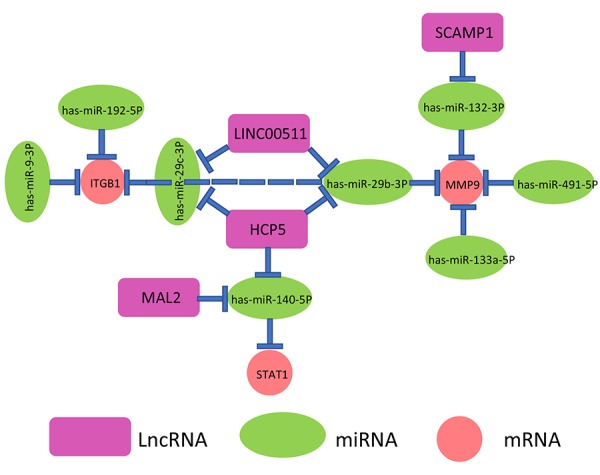
**The novel mRNA-miRNA-lncRNA competing endogenous RNA (ceRNA) triple regulatory network associated with prognosis of pancreatic cancer.**

By a series of *in silico* analyses, a key mRNA-miRNA-lncRNA competitive endogenous RNA triple regulatory network in pancreatic cancer were constructed. The network totally contained 9 mRNA-miRNA pairs (MMP9-miR-132-3p, MMP9-miR-133a-5p, MMP9-miR-29b-3p, MMP9-miR-491-5p, ITGB1-miR-192-5p, ITGB1-miR-29c-3p, ITGB1-miR-29b-3p, ITGB1-miR-9-3p and STAT1-miR-140-5p), 7 miRNA-lncRNA pairs (miR-132-3p-SCAMP1, miR-29b-3p-HCP5, miR-140-5p-HCP5, miR-29c-3p-HCP5, miR-140-5p-MAL2, miR-29b-3p-LINC00511 and miR-29c-3p-LINC00511) and 7 mRNA-lncRNA pairs (MMP9-SCAMP1, MMP9-LINC00511, MMP9-HCP5, ITGB1-HCP5, ITGB1-LINC00511, STAT1-HCP5 and STAT1-MAL2). This network was depicted in Figure 9.

As mentioned above, lncRNA can competitively bind to miRNA, thereby relieving suppressive effect of miRNA on mRNA. Based on this hypothesis, there are inverse relationships between miRNAs and lncRNAs or mRNAs and positive associations between mRNAs and lncRNAs. Herein, TCGA pancreatic cancer data were employed to determine the correlations of mRNA-miRNA or miRNA-lncRNA or mRNA-lncRNA pairs in the established network (Figure 9). As shown in Table 2, 6 of 9 mRNA-miRNA pairs (MMP9-miR-132-3p, MMP9-miR-29b-3p, MMP9-miR-491-5p, ITGB1-miR-192-5p, ITGB1-miR-29c-3p, ITGB1-miR-29b-3p), 3 of 7 miRNA-lncRNA pairs (miR-29b-3p-HCP5, miR-140-5p-MAL2 and miR-29c-3p-LINC00511) and 4 of 7 mRNA-lncRNA pairs (MMP9-HCP5, ITGB1-HCP5, STAT1-HCP5 and STAT1-MAL2) were fitted with the ceRNA mechanism. Taken all the three levels into consideration, we constructed a novel mRNA-miRNA-lncRNA triple sub-network, MMP9/ITGB1-miR-29b-3p-HCP5, which is significantly associated with prognosis of pancreatic cancer. The sub-network may also be developed as promising diagnostic biomarkers or therapeutic targets for pancreatic cancer in the future.

**Table 2:** The correlation between miRNA-mRNA pairs identified by starBase database (The pairs conformed to the ceRNA hypothesis are marked with Bold type).

miRNA	mRNA	R	P-value
**miR-132-3p**	**MMP9**	**-0.181**	**1.56e-02**
miR-133a-5p	MMP9	-0.048	5.22e-01
**miR-29b-3p**	**MMP9**	**-0.202**	**6.80e-03**
**miR-491-5p**	**MMP9**	**-0.194**	**9.48e-03**
**miR-192-5p**	**ITGB1**	**-0.223**	**2.80e-03**
**miR-29c-3p**	**ITGB1**	**-0.443**	**5.82e-10**
**miR-29b-3p**	**ITGB1**	**-0.440**	**7.78e-10**
miR-9-3p	ITGB1	0.096	2.03e-01
miR-140-5p	STAT1	0.016	8.34e-01
miRNA	lncRNA	R	P-value
miR-132-3p	SCAMP1	0.396	4.40e-08
**miR-29b-3p**	**HCP5**	**-0.163**	**3.00e-02**
miR-140-5p	HCP5	0.193	9.68e-03
miR-29c-3p	HCP5	0.009	9.01e-01
**miR-140-5p**	**MAL2**	**-0.204**	**6.25e-03**
miR-29b-3p	LINC00511	-0.051	4.99e-01
**miR-29c-3p**	**LINC00511**	**-0.219**	**3.34e-03**
mRNA	lncRNA	R	P-value
MMP9	SCAMP1	-0.129	8.57e-02
MMP9	LINC00511	0.061	4.16e-01
**MMP9**	**HCP5**	**0.267**	**3.15e-04**
**ITGB1**	**HCP5**	**0.223**	**2.77e-03**
ITGB1	LINC00511	0.046	5.45e-01
**STAT1**	**HCP5**	**0.591**	**3.67e-18**
**STAT1**	**MAL2**	**0.185**	**1.34e-02**

## DISCUSSION

Pancreatic cancer is notorious for its highly lethal nature and poor prognosis. Extremely poor prognosis of patients with pancreatic cancer greatly promotes us to develop effective treatment measures and excavate novel prognostic indicators. Only in these ways can the outcome of pancreatic cancer patients be improved rapidly. Recent studies have suggested that ncRNAs, including miRNAs and lncRNAs, play important roles in cancer initiation and progression [16–20]. After the first proposal of ceRNA hypothesis by Salmena *et al.* [4], increasing investigations regarding ceRNAs in human cancers have been carried out. For example, Liu *et al.* suggested that lncRNA XIST/miR-34a axis modulates thyroid cancer proliferation and growth by MET-PI3K-AKT signaling [21]; lncRNA XLOC_ 006390 was found to facilitate cervical cancer tumorigenesis and metastasis as a ceRNA against miR-331-3p and miR-338-3p [22]; Huang *et al.* found that H19 promoted non-small-cell lung cancer development by STAT3 signaling *via* sponging miR-17 [23]. In pancreatic cancer, many positive results have been also reported. Gao *et al.* demonstrated that lncRNA ZEB2-AS1 promoted pancreatic cancer cell growth and invasion by regulation of the miR-204/HMGB1 axis [24]; tumor-derived exosomal lncRNA SOX2OT was also observed to enhance EMT and stemness by acting as a ceRNA in pancreatic cancer [25]; Chen *et al.* indicated that lncRNA AFAP1-AS1 facilitated pancreatic cancer growth and invasion by upregulating the IGF1R oncogene through sequestration of miR-133a [26]; Gao *et al.* suggested that lncRNA ROR acted as a ceRNA to modulate Nanog expression by sponging miR-145 and predicted poor prognosis in pancreatic cancer [27]. However, integrated and comprehensive analysis of ceRNAs and mRNAs in pancreatic cancer is still not enough. To the best of our knowledge, this is the first study to investigate the specific ceRNA network in pancreatic cancer by way of “mRNA-miRNA-lncRNA” order pattern, instead of lncRNA-miRNA-mRNA order pattern. Inspiringly, a novel mRNA-miRNA-lncRNA triple regulatory network was constructed and each RNA in this network possessed a significant prognostic value in pancreatic cancer.

In this present study, we identified a total of 914 significant DEGs, consisting of 734 upregulated and 180 downregulated DEGs by intersection of DEGs from two GEO datasets, GSE16515 and GSE15471. GO is widely used as functional enrichment analysis for a large number of genes [28]. The results of these significant DEGs related GO analysis demonstrated that they were significantly enriched in some GO terms that were associated with cancer biological behaviors, including cell adhesion [29], wound healing [30] and activation of MAPK activity [31]. KEGG pathway enrichment analysis revealed that multiple enriched pathways were obtained, primarily involving pathways in cancer and metabolic pathways. Besides, GO analysis and pathway analysis also indicated that these significant DEGs were significantly enriched in focal adhesion. It has been well documented that focal adhesion and cell adhesion play key roles in cancer invasion and metastasis, thereby causing cancer progression [32,33]. Thus, these significant DEGs may be involved in modulation of invasion and metastasis of pancreatic cancer.

To systemically analyze the relationships and functions of significant DEGs in pancreatic cancer, we mapped the DEGs into STRING database and obtained PPI networks. A variety of interactions among these significant DEGs were obtained, especially for the upregulated significant DEGs. It has been widely acknowledged that genes with more node degree in the PPI network usually play more roles. Therefore, we screened the hub genes in the two PPI networks according to node degree. For further identifying key genes in pancreatic cancer, the top ten upregulated and downregulated hub genes were selected for further expression and survival analyses. The analytic results demonstrated that 7 upregulated (MMP9, CXCL8, ACTB, ITGB1, STAT1, TOP2A and CDK1) and 2 downregulated (GNMT and ABAT) hub genes may act as the key genes in pancreatic cancer. Intriguingly, most of these key genes have been well investigated in pancreatic cancer. For example, MMP9 participated in pancreatic cancer angiogenesis and invasion [34,35]; CXCL8 promoted invasiveness and angiogenesis in pancreatic cancer [36]; ITGB1 was upregulated in pancreatic cancer and increased ITGB1 indicated a poor outcome [37]; and STAT1 enhanced pancreatic cancer growth and metastasis [38]. These publications partially support the accuracy of our bioinformatic analyses.

MiRNAs and lncRNAs, are involved in regulation of gene expression and function by ceRNA mechanism as previously described. Some upstream miRNAs of the key genes were first predicted. Survival analysis revealed that patients with higher expression of 8 miRNAs (miR-132-3p, miR-133a-5p, miR-29b-3p, miR-491-5p, miR-192-5p, miR-29c-3p, miR-9-3p and miR-140-5p) have better prognosis in pancreatic cancer. The tumor suppressive roles of the 8 miRNAs in pancreatic cancer have been reported. For example, Abukiwan *et al*. suggested that inhibition of miR-132-3p drove progression of pancreatic cancer [39]; miR-133a-5p was also found to function as a tumor suppressor in pancreatic cancer [40,41]; the group of Wang Lihua showed that miR-29b-3p decreased proliferation and mobility of pancreatic cancer by targeting SOX12 and DNMT3b [42]. Then, we further predicted 90 upstream lncRNAs of these key miRNAs. By combining expression analysis and survival analysis for these lncRNAs in pancreatic cancer using TCGA data, only 4 lncRNAs (SCAMP1, HCP5, MAL2, LINC00511) were defined as the key lncRNAs. SCAPM1 suppressed migration and invasion of pancreatic cancer [43]; MAL2 expression predicted distant metastasis in pancreatic cancer [44]. Regarding to HCP5 and LINC00511, a variety of studies have also suggested that they act as two crucial oncogenes in human cancers [45,46]. Thus, a prognosis-associated mRNA-miRNA-lncRNA network in pancreatic cancer was successfully established. In this network, some pairs have been identified. For example, Lu *et al.* demonstrated that miR-29c inhibited pancreatic cancer cell growth, invasion and migration by targeting ITGB1 [47]. These reports further imply the accuracy of our current analytic results. Finally, correlation analysis for the RNA pairs in the constructed mRNA-miRNA-lncRNA network revealed that only MMP9/ITGB1-miR-29b-3p-HCP5 sub-network absolutely conformed to ceRNA hypothesis. Certainly, although attractive findings have been obtained by a series of bioinformatic analyses in our current study, more lab experiments and large-scale clinical trials need to be performed in the future.

## Conclusions

In summary, by integrated bioinformatics analysis, we constructed a novel mRNA-miRNA-lncRNA ceRNA triple regulatory network, in which all RNAs possessed significant predictive values for pancreatic cancer prognosis. In addition to the prognostic value of this mRNA-miRNA-lncRNA network in pancreatic cancer, it also provides some key clues for molecular mechanistic investigations of pancreatic cancer in the future. However, our team and other labs should conduct more studies to further validate these findings.

## MATERIALS AND METHODS

### MicroRNA microarray

At the first step, we searched for the datasets that compared gene expression between pancreatic cancer tissues and normal tissues in the Gene Expression Omnibus database (http://www.ncbi.nlm.nih.gov/geo/). Only datasets containing more than 15 cancer samples and 15 normal samples were included. Then, the titles and abstracts of these datasets were screened and full information of the datasets of interest were further evaluated. Finally, only two datasets (GSE16515 and GSE15471), based on the platform of Affymetrix Human Genome U133 Plus 2.0 Array (GPL570), were selected for subsequent analyses. GSE16515 dataset contained 36 pancreatic tumor samples and 16 normal samples, and GSE15471 dataset contained 36 pairs of pancreatic cancer tissues and adjacent normal tissues.

### Differential expression analysis

The online analytic tool GEO2R (https://www.ncbi.nlm.nih.gov/geo/geo2r), provided by the GEO database, was utilized to obtain DEGs from the two datasets. |log_2_FC| > 1 and adjusted p-value (adj. p-value) < 0.05 were set as the cut-off criteria when we performed the differential expression analysis. Besides, we employed an online tool, VENNY 2.1.0 (http://bioinfogp.cnb.csic.es/tools/venny/index.html), to draw the Venn diagrams. The DEGs that were commonly appeared in both GSE16515 and GSE15471 datasets were re-defined as the significant DEGs, including upregulated significant DEGs and downregulated significant DEGs.

### Gene ontology and KEGG pathway enrichment analysis

Database for Annotation, Visualization, and Integrated Discovery (https://david.ncifcrf.gov/) was introduced to conduct Gene Ontology (GO) functional annotation and Kyoto Encyclopedia of Genes and Genomes (KEGG) pathway enrichment analysis. The enriched GO terms and KEGG pathways were downloaded from the webpage. p-value < 0.05 was considered as statistically significant. Then, the top 10 enriched GO terms and KEGG pathways were displayed using ggplot2 package of R software [48].

### Protein-protein interaction (PPI) network

The PPI interaction networks between the DEGs were constructed by Search Tool for the Retrieval of Interacting Genes (STRING) database (http://string-db.org/) [49]. Firstly, the DEGs were typed into the database. Then, high-resolution bitmaps were displayed and downloaded from the webpage. Only these interactors with combined confidence score >= 0.4 were shown in the bitmap.

### Identification of hub genes

By calculating the degree of connectivity as we previously reported [50–52], the hub genes in the PPI networks were identified using CytoHubba, a plugin in Cytoscape software (Version 3.6.1). According to node degree, the top 30 hub genes were displayed in the Cytoscape software (Version 3.6.1).

### Gene expression analysis

In the TCGA project, there are only 4 pancreatic normal samples, which are too small sample size for performing the comparison between pancreatic cancer and normal controls. Gene Expression Profiling Interactive Analysis (GEPIA) (http://gepia.cancer-pku.cn/detail.php) is a newly developed interactive web server for analyzing the RNA sequencing expression data of 9736 tumors and 8587 normal samples from the TCGA and the GTEx projects [53]. In this study, GEPIA database, containing 179 pancreatic cancer samples and 171 normal samples, was used to analyze expression levels of key genes and lncRNAs in pancreatic cancer. Genes with |log_2_FC| > 1 and p-value < 0.05 were considered as statistically significant.

### Survival analysis

Prognostic values of genes, miRNAs and lncRNAs in pancreatic cancer were analyzed using Kaplan-Meier plotter database, which is capable to assess the effect of 54675 genes on survival using 10461 cancer samples [54]. Pancreatic cancer mRNA RNA-seq and miRNA data from “Pan-cancer” item in Kaplan-Meier plotter database were selected. These genes, miRNAs and lncRNAs were first entered into the database. Then, the hazard ratio (HR) with 95% confidence interval and logrank p-value were automatically calculated and directly displayed on the webpage. Logrank p-value < 0.05 was regarded as statistically significant.

### Prediction of miRNA

Upstream miRNAs of key genes were predicted using miRTarbase database [55]. In miRTarbase database, the collected microRNA-target interactions are experimentally validated by reporter assay, western blot, qPCR, microarray and next-generation sequencing experiments. To obtain more accurate prediction results, in this study, we only included microRNA-target interactions that were validated by reporter assay. Prognostic values of these predicted miRNAs were further assessed using Kaplan-Meier plotter database as mentioned above.

### Prediction of lncRNA

In this study, miRNet database was used to predict the upstream lncRNAs of miRNAs, which is a an easy-to-use tool for miRNA-associated studies [56,57]. “Organism-H.sapies”, “Tissue-Pancreas” and “target type-lncRNAs” were set as selection criteria.

### Correlation analysis

The correlations of mRNA-miRNA, miRNA-lncRNA and mRNA-lncRNA pairs in pancreatic cancer were evaluated using starBase database, which is an open-source platform for studying the ncRNA interactions from CLIP-seq, degradome-seq and RNA-RNA interactome data [58,59]. p-value < 0.05 was considered as statistically significant.

### Availability of data and materials

Please contact author for data and materials requests.

## SUPPLEMENTARY MATERIAL

Supplementary Table S1

Supplementary Table S2

Supplementary Table S3

Supplementary Table S4
