# Supplementary material for: A novel mRNA-miRNA-lncRNA competing endogenous RNA triple sub-network associated with prognosis of pancreatic cancer
Source: Aging (Albany NY). 2019 May 6;11(9):2610–27. doi: 10.18632/aging.101933 (PMC6535056; doi:10.18632/aging.101933)
Supplement: Supplementary Table S1 [file aging-11-101933-s001.docx]

**Table S1. The commonly upregulated genes in GSE16515 and GSE15471 datasets.**

| Upregulated genes |
| --- |
| S100P |
| CEACAM5 |
| CLDN18 |
| TRIM29 |
| CEACAM6 |
| TFF1 |
| KLK10 |
| SLC6A14 |
| CTSE |
| CXCL5 |
| DPCR1 |
| TMPRSS4 |
| KRT19 |
| SERPINB5 |
| GPRC5A |
| COL11A1 |
| MSLN |
| SFN |
| C19orf33 |
| SDR16C5 |
| MUC4 |
| LAMC2 |
| AGR2 |
| DKK1 |
| LCN2 |
| COL10A1 |
| REG4 |
| TSPAN1 |
| NQO1 |
| LAMB3 |
| AOC1 |
| CAPN8 |
| GABRP |
| GJB2 |
| PLAC8 |
| IL1RN |
| ZG16B |
| NMU |
| MMP11 |
| DUOX2 |
| IGFBP3 |
| SCEL |
| GCNT3 |
| CCL20 |
| IFI27 |
| TCN1 |
| AHNAK2 |
| SLPI |
| GPX2 |
| LAMA3 |
| KRT7 |
| POSTN |
| VSIG1 |
| ANXA10 |
| PHLDA2 |
| IGF2BP3 |
| TMC5 |
| S100A6 |
| CEACAM1 |
| HS3ST1 |
| INHBA |
| MMP12 |
| SFTA2 |
| FXYD3 |
| SLC2A1 |
| CXCL8 |
| CST1 |
| DHRS9 |
| ITGA2 |
| MMP1 |
| SLC44A4 |
| SERPINB2 |
| S100A2 |
| SULF1 |
| PI3 |
| ABHD17C |
| ACSL5 |
| GALNT5 |
| TMPRSS3 |
| TMEM45B |
| TFAP2A |
| ST6GALNAC1 |
| MTMR11 |
| EPPK1 |
| CEMIP |
| SGPP2 |
| MLPH |
| PLAU |
| CTHRC1 |
| HK2 |
| CDH3 |
| S100A14 |
| ECT2 |
| ANLN |
| XDH |
| CLDN23 |
| WFDC2 |
| LEMD1 |
| SDC1 |
| C15orf48 |
| FOXQ1 |
| MET |
| HN1 |
| SULT1C2 |
| KRT23 |
| ANKRD22 |
| ARNTL2 |
| MALL |
| LGALS4 |
| TOP2A |
| CRIP1 |
| FN1 |
| CD55 |
| STYK1 |
| COL12A1 |
| ADGRF1 |
| LY6E |
| COL8A1 |
| KLF5 |
| IFI6 |
| LEF1 |
| TSPAN8 |
| CDCA7 |
| KYNU |
| PLEK2 |
| PLAUR |
| DMBT1 |
| MMP7 |
| OAS1 |
| F5 |
| MXD1 |
| EGLN3 |
| PLPP4 |
| ECM1 |
| COL5A1 |
| ID1 |
| BIK |
| RRM2 |
| C1orf106 |
| KRT6B |
| KRT6A |
| KCNK1 |
| ITGB4 |
| SERPINB3 |
| NOX4 |
| CLDN4 |
| CXCL3 |
| FXYD5 |
| CP |
| MMP28 |
| EFNA5 |
| ANO1 |
| TACSTD2 |
| LOXL2 |
| TRIM31 |
| ISG15 |
| S100A11 |
| ITGA3 |
| TM4SF1 |
| CCNB1 |
| HOXC6 |
| CORO2A |
| AP1S3 |
| FERMT1 |
| S100A4 |
| ULBP2 |
| MX1 |
| SHISA2 |
| KCNN4 |
| OLR1 |
| WISP1 |
| CAPG |
| PCDH7 |
| MUC5B |
| AGR3 |
| NEK2 |
| TMEM158 |
| FHL2 |
| THBS2 |
| HEPH |
| BCL2A1 |
| EPSTI1 |
| ASPM |
| ISG20 |
| LYZ |
| ADAM9 |
| HOXB7 |
| ERO1A |
| COMP |
| GREM1 |
| VCAN |
| CAMK2N1 |
| COL1A1 |
| CDK1 |
| COL1A2 |
| IL1R2 |
| PDZK1IP1 |
| BHLHE40 |
| SLC9B2 |
| S100A16 |
| LRRC15 |
| CKS2 |
| PFKP |
| MAL2 |
| MARCKSL1 |
| MBOAT2 |
| TREM1 |
| DLGAP5 |
| NFE2L3 |
| OAS2 |
| RHBDL2 |
| IFI44L |
| PTTG1 |
| EFNB2 |
| MUC20 |
| KIAA1211 |
| MGLL |
| CENPF |
| MELK |
| CDH11 |
| HIST1H2BD |
| COL5A2 |
| INPP4B |
| EREG |
| CEP55 |
| ADAM28 |
| BAIAP2L1 |
| WNT2 |
| CCL18 |
| NUSAP1 |
| AREG |
| FAM83D |
| XAF1 |
| SQLE |
| CXCL10 |
| HOPX |
| RSAD2 |
| LY75 |
| CD109 |
| RUNX2 |
| APOL1 |
| TOX3 |
| EPYC |
| ANXA3 |
| TNFRSF21 |
| NPR3 |
| FAP |
| MX2 |
| PLS1 |
| SUCNR1 |
| ADAM12 |
| FOXL1 |
| LGALS3BP |
| SCIN |
| CENPU |
| ADAMTS12 |
| KIF26B |
| RTP4 |
| C1orf116 |
| CENPK |
| MYOF |
| ASPHD2 |
| SAMD9 |
| SOX4 |
| KIF14 |
| CLDN2 |
| HMGA2 |
| OSBPL3 |
| GPR87 |
| FGD6 |
| SLC6A6 |
| CEACAM7 |
| OAS3 |
| PTGS2 |
| TRNP1 |
| CYP3A5 |
| ADAMTS2 |
| HOXB3 |
| S100A10 |
| ELOVL6 |
| MXRA5 |
| PRC1 |
| OSBPL10 |
| SCD |
| ELF3 |
| TGM2 |
| C1GALT1 |
| SYTL2 |
| EDNRA |
| ITPR3 |
| PMEPA1 |
| PLAT |
| PKM |
| AMIGO2 |
| MMP9 |
| ANTXR1 |
| SULF2 |
| OPN3 |
| RAB31 |
| PLEC |
| PMAIP1 |
| COL8A2 |
| MMP14 |
| AEBP1 |
| NREP |
| EFNA1 |
| DTL |
| TNFAIP6 |
| HSD17B6 |
| CDKN2B |
| MAD2L1 |
| ENO2 |
| RHPN2 |
| RUNX1-IT1 |
| XRCC4 |
| NBL1 |
| CSTB |
| SERPINH1 |
| NHS |
| PSMB9 |
| IGFBP5 |
| TNFAIP2 |
| KRT8 |
| PDLIM7 |
| TRIM59 |
| HSPA6 |
| SRPX2 |
| TNFRSF11B |
| SEMA3C |
| UBE2S |
| GATA3 |
| IFI35 |
| SLC22A3 |
| TGFBI |
| DPYSL3 |
| BST2 |
| ZWINT |
| HOXA3 |
| MACROD2 |
| GOLM1 |
| VNN1 |
| PGK1 |
| BGN |
| FNDC1 |
| CXCR4 |
| EHF |
| NDNF |
| LPAR5 |
| SUGCT |
| IFI44 |
| STAT1 |
| IL1RAP |
| IFIT2 |
| PROM1 |
| DIO2 |
| LIF |
| ADGRG6 |
| TMEM173 |
| JUP |
| CD9 |
| MEGF6 |
| RARRES3 |
| CXCL14 |
| GRHL1 |
| SLC11A1 |
| ARL4C |
| LGALS3 |
| NCF2 |
| HILPDA |
| COL3A1 |
| ALOX5AP |
| PALLD |
| DPY19L1 |
| MELTF |
| PLD1 |
| FAM19A5 |
| CLIC1 |
| EDIL3 |
| NPC1 |
| DDX60 |
| SCNN1A |
| ARHGAP26 |
| LRRN1 |
| TPRG1 |
| PSMB8 |
| EPHA4 |
| PPP1R3B |
| GBP2 |
| RRAS |
| LOX |
| TIMP1 |
| VDR |
| BACE2 |
| DUSP5 |
| IFIT1 |
| SPARC |
| TPM2 |
| CERS6 |
| BASP1 |
| TAGLN2 |
| ARPC1B |
| PAQR8 |
| IFIT3 |
| TNFSF4 |
| NDC80 |
| C5orf46 |
| FSCN1 |
| ANXA2 |
| TMEM200A |
| PERP |
| RACGAP1 |
| PRR11 |
| KLF7 |
| CORIN |
| ANTXR2 |
| APOC1 |
| SLC16A6 |
| WDR72 |
| BICD1 |
| SLC12A2 |
| SPAG1 |
| ELF4 |
| NT5E |
| MPZL2 |
| WNT5A |
| LAMP5 |
| HPSE |
| IRAK2 |
| LBH |
| COTL1 |
| SLC16A1 |
| GBP1 |
| SMC4 |
| FAM83B |
| OLFML2B |
| CCNA2 |
| CD58 |
| ASAP2 |
| SLC16A4 |
| DACT1 |
| MSMO1 |
| PGM2L1 |
| TRIM14 |
| KIAA0930 |
| CKLF |
| GNA15 |
| CSTA |
| ANXA1 |
| TUBA4A |
| HLA-F |
| LDLR |
| SERPINA1 |
| STS |
| ENTPD1 |
| SKAP2 |
| NUAK1 |
| TNFSF11 |
| ITGBL1 |
| CTSK |
| CMTM3 |
| CDC42EP5 |
| APOE |
| FBXO32 |
| STEAP1 |
| TSPO |
| ENDOD1 |
| SLC39A10 |
| ZNF703 |
| TRIB2 |
| LGALS1 |
| SLC7A7 |
| BCL10 |
| PELI1 |
| MYL9 |
| CTTNBP2NL |
| FAT1 |
| GPR137B |
| ICAM1 |
| ALOX5 |
| SDC4 |
| ANXA2P2 |
| LCK |
| PRR5L |
| TPM4 |
| NCEH1 |
| SLC1A1 |
| ACTA2 |
| ISM1 |
| FOXC1 |
| PHLDA1 |
| SOX11 |
| CD47 |
| SLC2A3 |
| HRH1 |
| ITGB2 |
| PPL |
| MDK |
| CD300LF |
| GBP3 |
| PYCARD |
| C11orf80 |
| LAMA4 |
| RALA |
| TAP1 |
| MCUB |
| MAP4K4 |
| CALD1 |
| SOX9 |
| FCGR3B |
| MBOAT1 |
| ADAM10 |
| CENPW |
| NT5DC2 |
| C1QTNF3 |
| MFAP5 |
| KLF2 |
| GDA |
| FAR2 |
| RAC2 |
| TMEM133 |
| SHROOM3 |
| CAP1 |
| TMSB10 |
| DGKH |
| TRBC1 |
| SPOCK1 |
| TGIF1 |
| HCP5 |
| MATN3 |
| HOTAIRM1 |
| NMI |
| PXDN |
| LINC01094 |
| UBE2L6 |
| CTSA |
| KPNA2 |
| SESTD1 |
| ANOS1 |
| ASPH |
| TPBG |
| HOXB2 |
| RNF213 |
| CHST11 |
| RAPH1 |
| ENO1 |
| HBEGF |
| TFPI |
| ISLR |
| IER5L |
| PRDX1 |
| HOXB6 |
| ETV1 |
| RIT1 |
| MVP |
| LINC00342 |
| CTSB |
| PDLIM3 |
| BPGM |
| LDHA |
| YWHAZ |
| LOC400043 |
| SH3KBP1 |
| RAI14 |
| CST4 |
| MFAP2 |
| RASAL2 |
| SLC24A3 |
| PLA2G7 |
| CST2 |
| PTBP3 |
| PON2 |
| IFI16 |
| CTSS |
| SPATS2L |
| CD52 |
| TWIST1 |
| SLCO3A1 |
| APLP2 |
| FZD2 |
| B4GALT5 |
| AHR |
| SMYD3 |
| PRRX1 |
| LOXL1 |
| TGFBR1 |
| HK1 |
| CLDN1 |
| TGFB1 |
| DDX60L |
| NET1 |
| ITGB5 |
| PLA2R1 |
| DKK3 |
| ADAMTS6 |
| PARP9 |
| SLC44A1 |
| ID3 |
| DOCK5 |
| HLA-B |
| ZNF532 |
| ABCG1 |
| LAYN |
| MREG |
| COL4A1 |
| CD86 |
| TNIK |
| SAT1 |
| LMO4 |
| RAP2B |
| EGFL6 |
| CORO1C |
| EMB |
| GALNT10 |
| ERI1 |
| CFL1 |
| NEK6 |
| KITLG |
| HAVCR2 |
| PRKCI |
| NAV1 |
| MPZL1 |
| DCBLD1 |
| LAPTM5 |
| FOSL2 |
| RNF145 |
| ONECUT2 |
| LRRFIP1 |
| CKAP2 |
| FBN1 |
| EIF5A2 |
| TNFRSF10A |
| SAMD9L |
| PTPN12 |
| ITGB1 |
| TGFB1I1 |
| ACTN1 |
| STX7 |
| COL16A1 |
| EPS8 |
| PIEZO1 |
| CALU |
| ABRACL |
| PPP1R18 |
| SMURF1 |
| CFB |
| MICAL2 |
| SERPINB9 |
| KIAA1217 |
| TUBA1C |
| DCBLD2 |
| GPX8 |
| SRGAP2C |
| NRP2 |
| ETS2 |
| GINS1 |
| STAMBPL1 |
| PTPRC |
| N4BP1 |
| DUSP6 |
| PLEKHG1 |
| HENMT1 |
| MFGE8 |
| TAP2 |
| ZNF267 |
| TYROBP |
| C1QB |
| LPCAT2 |
| B3GNT5 |
| THAP9-AS1 |
| DLEU2 |
| LOC105375172 |
| AMMECR1 |
| IER3 |
| MYO1E |
| ARHGDIB |
| GPNMB |
| SMS |
| MXRA8 |
| MYLK |
| IFITM1 |
| ITGAM |
| CMTM6 |
| THY1 |
| MAP3K8 |
| PRDM1 |
| CTSC |
| TAF9B |
| CHML |
| FGD5 |
| ZNF365 |
| TIGAR |
| ABR |
| HLA-A |
| ATP1B3 |
| UHMK1 |
| PDLIM4 |
| CNIH4 |
| DST |
| PRRG1 |
| SPP1 |
| CASP1 |
| RGS10 |
| IGSF6 |
| KDM5B |
| SIPA1L2 |
| PNMA1 |
| MAFF |
| IRF9 |
| VCL |
| TUBA1B |
| KDELC1 |
| CD300A |
| DLG1 |
| SLFN5 |
| TAGLN |
| ACTB |
| TREM2 |
| RND3 |
| SPTLC2 |
| PFN1 |
| FOXF2 |
| TNFSF10 |
| H2AFY |
| SYT13 |
| SGMS2 |
| RGS1 |
| IER5 |
| GLI2 |
| OSMR |
| UBA6 |
| CPNE8 |
| FLNA |
| COL6A3 |
| PDPN |
