# Supplementary material for: A novel mRNA-miRNA-lncRNA competing endogenous RNA triple sub-network associated with prognosis of pancreatic cancer
Source: Aging (Albany NY). 2019 May 6;11(9):2610–27. doi: 10.18632/aging.101933 (PMC6535056; doi:10.18632/aging.101933)
Supplement: Supplementary Table S2 [file aging-11-101933-s002.docx]

**Table S3. The miRNA-mRNA pairs predicted by miRTarBase database.**

| mRNA | miRNA |
| --- | --- |
| MMP9 | hsa-let-7e-5p |
| MMP9 | hsa-miR-132-3p |
| MMP9 | hsa-miR-133a-5p |
| MMP9 | hsa-miR-133b |
| MMP9 | hsa-miR-204-5p |
| MMP9 | hsa-miR-29b-3p |
| MMP9 | hsa-miR-491-5p |
| CXCL8 | hsa-miR-100-3p |
| CXCL8 | hsa-miR-106a-5p |
| CXCL8 | hsa-miR-23a-3p |
| CXCL8 | hsa-miR-302c-3p |
| CXCL8 | hsa-miR-302d-3p |
| CXCL8 | hsa-miR-520b |
| CXCL8 | hsa-miR-93-5p |
| ACTB | hsa-miR-644a |
| ITGB1 | hsa-miR-124-3p |
| ITGB1 | hsa-miR-130b-3p |
| ITGB1 | hsa-miR-134-5p |
| ITGB1 | hsa-miR-183-5p |
| ITGB1 | hsa-miR-192-5p |
| ITGB1 | hsa-miR-29a-3p |
| ITGB1 | hsa-miR-29b-3p |
| ITGB1 | hsa-miR-29c-3p |
| ITGB1 | hsa-miR-9-3p |
| STAT1 | hsa-miR-140-5p |
| STAT1 | hsa-miR-145-5p |
| STAT1 | hsa-miR-146a-5p |
| STAT1 | hsa-miR-150-5p |
| STAT1 | hsa-miR-155-5p |
| STAT1 | hsa-miR-203a-3p |
| STAT1 | hsa-miR-223-3p |
| STAT1 | hsa-miR-450a-5p |
| CDK1 | hsa-miR-24-3p |
| CDK1 | hsa-miR-31-5p |
