# Supplementary material for: A novel mRNA-miRNA-lncRNA competing endogenous RNA triple sub-network associated with prognosis of pancreatic cancer
Source: Aging (Albany NY). 2019 May 6;11(9):2610–27. doi: 10.18632/aging.101933 (PMC6535056; doi:10.18632/aging.101933)
Supplement: Supplementary Table S3 [file aging-11-101933-s003.docx]

**Table S4. The lncRNA-miRNA pairs predicted by miRNet database.**

| miRNA | lncRNA |
| --- | --- |
| hsa-mir-29b-3p | AC005154.5 |
| hsa-mir-29c-3p | AC005154.5 |
| hsa-mir-29b-3p | AC005154.6 |
| hsa-mir-29c-3p | AC005154.6 |
| hsa-mir-29b-3p | AC007036.5 |
| hsa-mir-29c-3p | AC007036.5 |
| hsa-mir-29b-3p | AC012146.7 |
| hsa-mir-29c-3p | AC012146.7 |
| hsa-mir-132-3p | AC012307.3 |
| hsa-mir-140-5p | AC058791.1 |
| hsa-mir-140-5p | AC079117.1 |
| hsa-mir-192-5p | AC084018.1 |
| hsa-mir-29b-3p | AP000304.2 |
| hsa-mir-29c-3p | AP000304.2 |
| hsa-mir-132-3p | COX10-AS1 |
| hsa-mir-132-3p | CTA-204B4.6 |
| hsa-mir-140-5p | CTB-25J19.9 |
| hsa-mir-140-5p | CTC-459F4.3 |
| hsa-mir-29b-3p | CTD-2116N17.1 |
| hsa-mir-29c-3p | CTD-2116N17.1 |
| hsa-mir-29b-3p | CTD-2339L15.1 |
| hsa-mir-29c-3p | CTD-2339L15.1 |
| hsa-mir-140-5p | CTD-2369P2.2 |
| hsa-mir-29b-3p | CTD-2517M14.5 |
| hsa-mir-29c-3p | CTD-2517M14.5 |
| hsa-mir-140-5p | DHRS4-AS1 |
| hsa-mir-29b-3p | EMG1 |
| hsa-mir-29c-3p | EMG1 |
| hsa-mir-29b-3p | GAS5 |
| hsa-mir-29c-3p | GAS5 |
| hsa-mir-29b-3p | GS1-124K5.11 |
| hsa-mir-29c-3p | GS1-124K5.11 |
| hsa-mir-29b-3p | H19 |
| hsa-mir-140-5p | H19 |
| hsa-mir-29c-3p | H19 |
| hsa-mir-29b-3p | HCP5 |
| hsa-mir-140-5p | HCP5 |
| hsa-mir-29c-3p | HCP5 |
| hsa-mir-132-3p | HNRNPU-AS1 |
| hsa-mir-29b-3p | HOXA-AS3 |
| hsa-mir-29c-3p | HOXA-AS3 |
| hsa-mir-29b-3p | HOXA-AS4 |
| hsa-mir-29c-3p | HOXA-AS4 |
| hsa-mir-132-3p | ILF3-AS1 |
| hsa-mir-29b-3p | KCNQ1OT1 |
| hsa-mir-140-5p | KCNQ1OT1 |
| hsa-mir-29c-3p | KCNQ1OT1 |
| hsa-mir-132-3p | LA16c-313D11.11 |
| hsa-mir-29b-3p | LIFR-AS1 |
| hsa-mir-29c-3p | LIFR-AS1 |
| hsa-mir-132-3p | LINC00176 |
| hsa-mir-29b-3p | LINC00338 |
| hsa-mir-140-5p | LINC00338 |
| hsa-mir-29c-3p | LINC00338 |
| hsa-mir-29b-3p | LINC00511 |
| hsa-mir-29c-3p | LINC00511 |
| hsa-mir-140-5p | LINC00667 |
| hsa-mir-140-5p | MAL2 |
| hsa-mir-140-5p | MALAT1 |
| hsa-mir-29b-3p | MIAT |
| hsa-mir-29c-3p | MIAT |
| hsa-mir-29b-3p | MLLT4-AS1 |
| hsa-mir-29c-3p | MLLT4-AS1 |
| hsa-mir-29b-3p | OIP5-AS1 |
| hsa-mir-140-5p | OIP5-AS1 |
| hsa-mir-29c-3p | OIP5-AS1 |
| hsa-mir-140-5p | PRKCQ-AS1 |
| hsa-mir-140-5p | RP11-102F4.3 |
| hsa-mir-29b-3p | RP11-145M9.4 |
| hsa-mir-29c-3p | RP11-145M9.4 |
| hsa-mir-140-5p | RP11-159D12.9 |
| hsa-mir-192-5p | RP11-170L3.8 |
| hsa-mir-132-3p | RP11-197P3.5 |
| hsa-mir-29b-3p | RP11-216F19.2 |
| hsa-mir-192-5p | RP11-216F19.2 |
| hsa-mir-132-3p | RP11-216F19.2 |
| hsa-mir-29c-3p | RP11-216F19.2 |
| hsa-mir-29b-3p | RP11-220I1.1 |
| hsa-mir-29c-3p | RP11-220I1.1 |
| hsa-mir-29b-3p | RP11-227G15.3 |
| hsa-mir-29c-3p | RP11-227G15.3 |
| hsa-mir-192-5p | RP11-276H19.2 |
| hsa-mir-132-3p | RP11-277L2.2 |
| hsa-mir-140-5p | RP11-27I1.2 |
| hsa-mir-29c-3p | RP11-280F2.2 |
| hsa-mir-29b-3p | RP11-290F20.1 |
| hsa-mir-29c-3p | RP11-290F20.1 |
| hsa-mir-29b-3p | RP11-303E16.8 |
| hsa-mir-29c-3p | RP11-303E16.8 |
| hsa-mir-132-3p | RP11-305N23.1 |
| hsa-mir-29b-3p | RP11-311C24.1 |
| hsa-mir-29c-3p | RP11-311C24.1 |
| hsa-mir-29b-3p | RP11-347I19.3 |
| hsa-mir-192-5p | RP11-347I19.3 |
| hsa-mir-29c-3p | RP11-347I19.3 |
| hsa-mir-140-5p | RP11-350F4.2 |
| hsa-mir-29b-3p | RP11-373L24.1 |
| hsa-mir-29c-3p | RP11-373L24.1 |
| hsa-mir-192-5p | RP11-400F19.6 |
| hsa-mir-132-3p | RP11-403I13.4 |
| hsa-mir-29b-3p | RP11-429J17.2 |
| hsa-mir-29c-3p | RP11-429J17.2 |
| hsa-mir-29b-3p | RP11-467L20.9 |
| hsa-mir-29c-3p | RP11-467L20.9 |
| hsa-mir-132-3p | RP11-473I1.10 |
| hsa-mir-140-5p | RP11-473I1.10 |
| hsa-mir-29b-3p | RP11-480D4.3 |
| hsa-mir-29c-3p | RP11-480D4.3 |
| hsa-mir-140-5p | RP11-4F5.2 |
| hsa-mir-29b-3p | RP11-615I2.7 |
| hsa-mir-29c-3p | RP11-615I2.7 |
| hsa-mir-29b-3p | RP11-618G20.1 |
| hsa-mir-29c-3p | RP11-618G20.1 |
| hsa-mir-132-3p | RP11-64K12.2 |
| hsa-mir-140-5p | RP11-690D19.3 |
| hsa-mir-29b-3p | RP11-690G19.3 |
| hsa-mir-132-3p | RP11-690G19.3 |
| hsa-mir-29c-3p | RP11-690G19.3 |
| hsa-mir-140-5p | RP11-98D18.9 |
| hsa-mir-140-5p | RP13-507I23.1 |
| hsa-mir-29b-3p | RP4-665N4.8 |
| hsa-mir-29c-3p | RP4-665N4.8 |
| hsa-mir-192-5p | RP5-1028K7.2 |
| hsa-mir-29b-3p | RP5-837J1.2 |
| hsa-mir-29c-3p | RP5-837J1.2 |
| hsa-mir-140-5p | SBF2-AS1 |
| hsa-mir-132-3p | SCAMP1 |
| hsa-mir-29b-3p | SETD5-AS1 |
| hsa-mir-29c-3p | SETD5-AS1 |
| hsa-mir-29b-3p | SIK3-IT1 |
| hsa-mir-29c-3p | SIK3-IT1 |
| hsa-mir-140-5p | SNHG1 |
| hsa-mir-132-3p | SNHG16 |
| hsa-mir-140-5p | SNHG16 |
| hsa-mir-132-3p | SNHG5 |
| hsa-mir-29b-3p | SPPL2B |
| hsa-mir-29c-3p | SPPL2B |
| hsa-mir-29b-3p | TUG1 |
| hsa-mir-132-3p | TUG1 |
| hsa-mir-29c-3p | TUG1 |
| hsa-mir-29b-3p | U47924.19 |
| hsa-mir-29c-3p | U47924.19 |
| hsa-mir-29b-3p | XIST |
| hsa-mir-192-5p | XIST |
| hsa-mir-132-3p | XIST |
| hsa-mir-140-5p | XIST |
| hsa-mir-29c-3p | XIST |
| hsa-mir-29b-3p | ZNF518A |
| hsa-mir-29c-3p | ZNF518A |
| hsa-mir-29b-3p | ZNF761 |
| hsa-mir-29c-3p | ZNF761 |
| hsa-mir-132-3p | ZNRD1-AS1 |
