# Supplementary material for: A novel mRNA-miRNA-lncRNA competing endogenous RNA triple sub-network associated with prognosis of pancreatic cancer
Source: Aging (Albany NY). 2019 May 6;11(9):2610–27. doi: 10.18632/aging.101933 (PMC6535056; doi:10.18632/aging.101933)
Supplement: Supplementary Table S4 [file aging-11-101933-s004.docx]

**Table S2. The commonly downregulated genes in GSE16515 and GSE15471 datasets.**

| Downregulated genes |
| --- |
| CPEB3 |
| RAB3D |
| HRASLS5 |
| NRG4 |
| TRHDE-AS1 |
| CHRM3 |
| MTUS2 |
| P4HB |
| FAM46C |
| SLC25A15 |
| ALDH6A1 |
| STC2 |
| PPID |
| PBLD |
| SLC25A34 |
| A1CF |
| ENPP1 |
| LOC100289094 |
| PEBP4 |
| NRTN |
| KCNJ5 |
| PLCB1 |
| KCNJ16 |
| GCAT |
| MCOLN3 |
| CBARP |
| GUCA1C |
| ARSE |
| SOX15 |
| F11 |
| LOC101929726 |
| CYB5A |
| REEP1 |
| PIWIL2 |
| DDC |
| SLC35G1 |
| CBFA2T3 |
| SFTPC |
| ECI2 |
| CA4 |
| SPAG4 |
| RNF186 |
| EPOR |
| KIRREL2 |
| GMNN |
| RNASE1 |
| CRAT |
| GPT2 |
| SEC11C |
| MAT1A |
| LIFR |
| SLC41A1 |
| MPP7 |
| CCDC69 |
| PRDX4 |
| LOC100506691 |
| SLC25A45 |
| MYCL |
| SEL1L |
| ZG16 |
| IL22RA1 |
| HPN |
| IMPA2 |
| PTGER4 |
| PSAT1 |
| SLC39A8 |
| LOC100129129 |
| TCEA3 |
| PLCE1 |
| GPHA2 |
| GLS2 |
| ACAT1 |
| BTG2 |
| SIDT2 |
| TMEM97 |
| ALB |
| RAB26 |
| EPB41L4B |
| ATP4A |
| KLB |
| GATM |
| PEX5L |
| LPAR3 |
| DPP10-AS1 |
| PRLR |
| DMD |
| SLC39A5 |
| BRSK2 |
| SEMA6D |
| EPHX2 |
| LINC00261 |
| FKBP11 |
| FAM129A |
| NUCB2 |
| CTH |
| LOC285097 |
| LOC283075 |
| SLC1A2 |
| KCNK3 |
| ABAT |
| GSTA3 |
| LOC102723493 |
| SLC17A4 |
| VEPH1 |
| ECHDC3 |
| TDH |
| SERPINI1 |
| GAMT |
| C5 |
| CTNND2 |
| GAS2 |
| LOC101930067 |
| BACE1 |
| F8 |
| BNIP3 |
| MT1G |
| PDZK1 |
| TRIM50 |
| TPST2 |
| EPHX1 |
| IGFBP2 |
| PM20D1 |
| DPP10 |
| SLC30A2 |
| RBM20 |
| SLC16A10 |
| SLC4A4 |
| COCH |
| CCKBR |
| SLC43A1 |
| PAK3 |
| HOMER2 |
| CCDC110 |
| CBS |
| ACADL |
| SYBU |
| RGN |
| KIAA1324 |
| LINC00339 |
| DPEP1 |
| NR5A2 |
| TEX11 |
| TMEM52 |
| PRSS3 |
| MYRIP |
| MT1M |
| AOX1 |
| P2RX1 |
| PDK4 |
| LGALS2 |
| GP2 |
| ANPEP |
| TRHDE |
| LMO3 |
| ERO1B |
| DNASE1 |
| PAIP2B |
| GSTA1 |
| EGF |
| FGL1 |
| GNMT |
| RBPJL |
| IAPP |
| SPX |
| BHLHA15 |
| KLK1 |
| FAM150B |
| ERP27 |
| TMED6 |
| AQP8 |
| CELA2B |
| PDIA2 |
| CUZD1 |
| SYCN |
| PNLIPRP1 |
| CTRL |
| CPA2 |
| SERPINI2 |
| CELP |
| PNLIPRP2 |
